# Prevalence and Structured Approach to the Diagnoses of A on V Tachycardias

**DOI:** 10.1002/joa3.70329

**Published:** 2026-04-05

**Authors:** Shuta Kanai, Koichi Nagashima, Mitsunori Maruyama, Akihiko Nogami, Yosuke Nakatani, Hitoshi Hirano, Akira Kimata, Tatsuya Hayashi, Kentaro Hayashi, Masato Okada, Naoko Miyazaki, Akinobu Mizutani, Mebae Mizutani, Masafumi Kato, Hitoshi Mori, Satoshi Higuchi, Shohei Kataoka, Ryuta Watanabe, Shu Hirata, Masanaru Sawada, Yuji Saito, Hikaru Masuda, Yasuo Okumura, Melvin M. Scheinman

**Affiliations:** ^1^ Division of Cardiology, Department of Medicine Nihon University School of Medicine Tokyo Japan; ^2^ Department of Cardiovascular Medicine Nippon Medical School Musashikosugi Hospital Kawasaki Japan; ^3^ Department of Cardiology Tokyo Heart Rhythm Hospital Tokyo Japan; ^4^ Department of Cardiovascular Medicine Gunma University Graduate School of Medicine Maebashi Japan; ^5^ Department of Cardiology Tokyo Metropolitan Bokutoh Hospital Tokyo Japan; ^6^ Department of Cardiology, Faculty of Medicine University of Tsukuba Tsukuba Japan; ^7^ Division of Cardiovascular Medicine Saitama Medical Center, Jichi Medical University Saitama Japan; ^8^ Department of Cardiology Ageo Central General Hospital Ageo Japan; ^9^ Cardiovascular Center, Sakurabashi Watanabe Advanced Healthcare Hospital Osaka Japan; ^10^ Division of Cardiology Nagai Hospital Tsu Japan; ^11^ Department of Cardiology Saitama Medical University International Medical Center Saitama Japan; ^12^ Department of Cardiology Tokyo Women's Medical University Tokyo Japan; ^13^ Division of Cardiology, Section of Cardiac Electrophysiology University of California San Francisco San Francisco California USA

**Keywords:** A on V tachycardia, atrial tachycardia, atrioventricular nodal reentrant tachycardia, junctional tachycardia, nodofascicular pathway, nodoventricular pathway, orthodromic reciprocating tachycardia

## Abstract

**Background:**

“A on V” tachycardia (AoV‐T) is classically considered suggestive of atrioventricular nodal reentrant tachycardia (AVNRT); emerging evidence suggests that other mechanisms may present with this electrogram pattern.

**Objective:**

To reassess the prevalence and electrophysiologic characteristics of nonconventional mechanisms underlying AoV‐T.

**Methods:**

We retrospectively reviewed 1484 consecutive electrophysiologic studies for narrow‐QRS tachycardia with His–atrial interval ≤ 70 ms at 10 centers (2015–2024). Diagnoses were adjudicated using atrial overdrive pacing (AOP), ventricular overdrive pacing (VOP), and His‐synchronized premature ventricular contractions (PVCs).

**Results:**

Mechanisms other than conventional AVNRT were identified in 24 patients (1.6%): atrial tachycardia (AT, *n* = 7), junctional tachycardia (JT, *n* = 6), orthodromic reciprocating tachycardia via an NV pathway (NV‐ORT, *n* = 6) or an NF pathway (NF‐ORT, *n* = 1), and AVNRT with a bystander NV pathway (*n* = 4). AT arose from the crista terminalis (*n* = 4) or para‐Hisian region (*n* = 3) and was diagnosed by V‐A‐A‐V response following VOP and/or absence of VA linking during differential AOP; dual AV nodal physiology was frequently observed. JT was diagnosed with atrial extrastimulus and/or A‐H‐H‐A response after AOP. NV/NF‐ORT was diagnosed by combined findings, including tachycardia reset/termination with His‐refractory PVCs, short PPI – TCL, orthodromic His capture, and V‐V‐A response following VOP; QRS fusion favored NV‐ORT. AVNRT with a bystander NV pathway was distinguished from NV‐ORT by pacing responses consistent with AVNRT despite evidence of NV conduction.

**Conclusion:**

Although AVNRT predominates in AoV‐T, a small subset of cases reflects AT, JT, NV/NF‐ORT, or AVNRT with a bystander NV pathway. Comprehensive pacing maneuvers are essential to avoid misclassification.

## Graphical abstract

Schematic tachycardia circuit diagram and corresponding laddergram depicting how atrial and ventricular activations become coincident to exhibit true A on V tachycardia, contrasted with the electrophysiologic basis of pseudo A on V patterns.

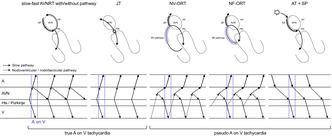

**Abbreviations**
AH: atrio‐His interval
AOP: atrial overdrive pacing
AP: accessory pathway
AT: atrial tachycardia
AVNRT: atrioventricular nodal reentrant tachycardia
HA: His–atrial interval
HV: His–ventricular interval
JT: junctional tachycardia
NF: nodofascicular pathway
NV: nodoventricular pathway
ORT: orthodromic reciprocating tachycardia
PCL: pacing cycle length
PPI: post‐pacing interval
PVC: premature ventricular contraction
RV: right ventricle/right ventricular
SP: slow pathway
TCL: tachycardia cycle length
VA: ventriculo‐atrial
VOP: ventricular overdrive pacing

## 1 Introduction

Advances in diagnostic maneuvers have facilitated the identification of novel mechanisms underlying supraventricular tachycardia (SVT) [1, 2]. In characterizing SVT, the term “A on V” tachycardia is used when atrial electrograms are inscribed on or very close to the ventricular electrograms in the septal region, and it is often described as a ventriculoatrial (VA) interval of < 70 ms in adults [3]. Although “A on V” tachycardia has traditionally been associated with atrioventricular nodal reentrant tachycardia (AVNRT) [4], recent reports have highlighted several additional mechanisms, including junctional tachycardia (JT), atrial tachycardia (AT), and orthodromic reciprocating tachycardia (ORT) via a nodoventricular/nodofascicular (NV/NF) pathway [2, 5, 6, 7]. However, the prevalence and specific diagnostic criteria for these exceptions have not been well clarified. Therefore, this study aims to re‐evaluate the concept of “A on V” tachycardia by investigating the prevalence of each underlying mechanism that exhibits this electrogram pattern.

## 2 Methods

### 2.1 Study Patients

We asked 10 clinical centers to retrospectively review electrophysiologic studies and/or catheter ablation procedures performed for A on V tachycardia, defined as a narrow QRS tachycardia with a His–atrial (HA) interval ≤ 70 ms, between 2015 and 2024 [3, 8]. In total, 1484 studies from 1484 patients were reviewed. The mean review period at each participating center was 6.5 ± 2.4 years.

### 2.2 Electrophysiologic Study

An electrophysiologic study was performed with patients under conscious sedation. Four‐ to 10‐pole electrode catheters were placed in the high right atrium (HRA), right ventricular (RV) apex, and His‐bundle region through the right femoral vein. Additionally, a 10‐ or 20‐pole electrode catheter was placed in the coronary sinus (CS) via the right jugular vein. Bipolar intracardiac electrograms were recorded through a band‐pass filter of 30 to 500 Hz at paper speeds ranging from 100 to 200 mm/s and stored on a digital recording system (LabSystem PRO, Bard Electrophysiology; or CardioLab EP, GE Healthcare). Bipolar pacing was performed at a current strength of 10 mA and a pulse width of 2 ms. Isoproterenol was administered if the tachycardia was not inducible or atrio‐His (AH) block, VA block, or a change in the VA interval was seen during the tachycardia. Diagnostic pacing maneuvers were preferably attempted when the tachycardia with a stable 1:1 VA relationship was confirmed; however, if that was not possible, pacing was performed even in the absence of a 1:1 VA relationship.

The A on V tachycardia was further classified as either an H‐A‐V sequence or an H‐V‐A sequence. The H‐A‐V sequence was defined as a sequence in which the earliest atrial electrogram was identified between the His electrogram and QRS onset, whereas the H‐V‐A sequence was defined as a sequence in which the earliest atrial electrogram was identified after QRS onset.

### 2.3 Diagnostic Pacing Maneuvers

Each diagnosis was made according to the following criteria. The choice of diagnostic pacing maneuvers was at the discretion of each institution or physician, but VOP was performed in all cases.

AT was diagnosed on the basis of the following criteria: (1) resumption of the tachycardia with a V‐A‐A‐V response after ventricular overdrive pacing (VOP) in the presence of VA conduction [1]; and (2) absence of VA linking, indicated by a ΔVA interval of > 20 ms following differential atrial overdrive pacing (AOP) from HRA, proximal CS and distal CS in the absence of VA conduction [1, 9].

JT was diagnosed on the basis of one or more of the following criteria: (1) resumption of the tachycardia with an A‐H‐H‐A response after AOP [10], (2) absence of tachycardia resetting immediately following a late coupled atrial extrastimulus, which failed to reset the His electrogram, and (3) sustained tachycardia following an early coupled atrial extrastimulus, which captured immediately following the His electrogram [11].

With AT and JT excluded by the absence of the above criteria, the final step was to differentiate ORT via an NV/NF pathway from AVNRT. Atrial activation and ventricular activation cannot occur simultaneously in ORT via an atrioventricular accessory pathway. ORT via an NV/NF pathway was diagnosed on the basis of the combination of criterion (1) and at least one of criteria (2–5): (1) Advancement or delay of His‐bundle timing by ≥ 10 ms, or termination of the tachycardia without atrial capture, upon delivery of scanned single premature ventricular contractions (PVC) during His‐bundle refractoriness or within the transition zone of VOP [1, 12], (2) a V‐V‐A response due to an extremely long stimulus‐atrial (SA) interval exceeding the tachycardia cycle length (TCL); (3) an uncorrected/corrected post‐pacing interval (PPI) minus a TCL of ≤ 125 ms/≤ 110 ms [13], and (4) orthodromic His or septal ventricular capture during VOP [14], and (5) a paradoxical AH response, defined as an AH interval during SVT shorter than that during sinus rhythm, or a > 40‐ms change in the AH interval when comparing SVT with the AH interval during AOP at the same pacing cycle length (PCL) as the TCL [15]. Moreover, the presence of constant/progressive QRS fusion helps to distinguish NV‐ORT from NF‐ORT [16]. Constant/progressive QRS fusion is absent in cases of NF‐ORT because the circuit is confined to the His‐Purkinje system and does not involve ventricular myocardium.

AVNRT with a bystander NV pathway was considered when only criterion (1) was present in the absence of criteria (2–5). In this situation, ORT was excluded by demonstrating dissociation of the His‐bundle from the tachycardia, evidenced by AH block during tachycardia or disappearance of the tachycardia‐resetting phenomenon after delivery of an early PVC [2]. Notably, reset or termination of the tachycardia without atrial capture occurring one cycle after a His‐refractory PVC was considered specifically diagnostic of AVNRT with a bystander NV pathway [1, 2] After establishing this diagnosis, the next step was to determine the AVN insertion site of the NV pathway according to the reset/termination sequence: when atrial reset or block preceded His reset/block after a His‐refractory PVC, the NV pathway was considered to insert into the retrograde limb of the AVNRT circuit; conversely, when His reset/block preceded atrial reset/block, insertion into the anterograde limb was presumed [2].

If none of the above criteria were present, the AVNRT was diagnosed. AOP and VOP were initiated when the coupling interval from the last catheter‐sensed atrial or RV electrogram to the first paced beat approximated the PCL, which was generally set 10–30 ms shorter than the TCL.

### 2.4 Statistical Analyses

Data are shown as the number (and percentage) of patients, or mean ± SD values.

## 3 Results

### 3.1 Non‐AVNRT Mechanisms Underlying A on V Tachycardia

**FIGURE 1:**
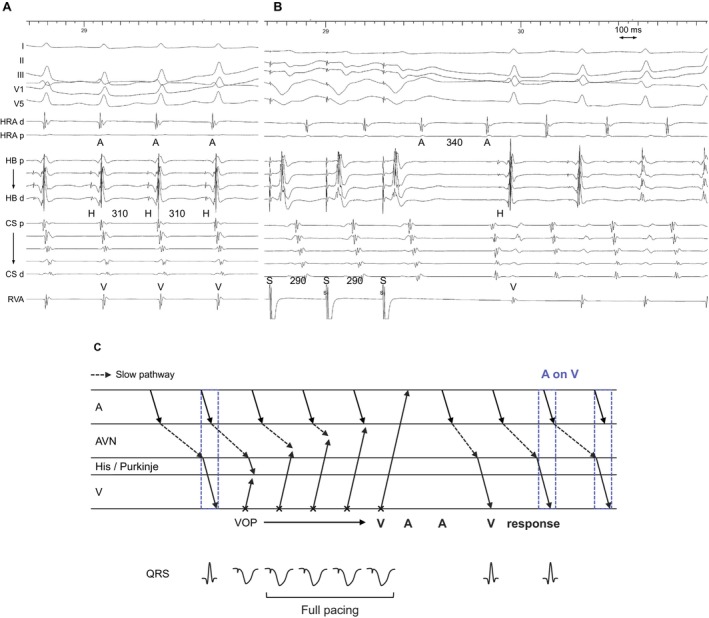
(A) Intracardiac electrograms of atrial tachycardia (AT) and (B) ventricular overdrive pacing (VOP) during AT. Immediately after VOP, the AH interval shortens but then progressively prolongs, ultimately returning to A on V tachycardia. (C) Schematic laddergram of VOP illustrating the A on V mechanism and a V‐A‐A‐V response. This laddergram is not intended to exactly match the intracardiac electrograms shown in (B). A = atrium, AH = atrio‐His, CS = coronary sinus, d = distal, H = His, HB = His bundle, HRA = high right atrium, *p* = proximal, RVA = right ventricular apex, S = stimulus, V = ventricle.

**FIGURE 2:**
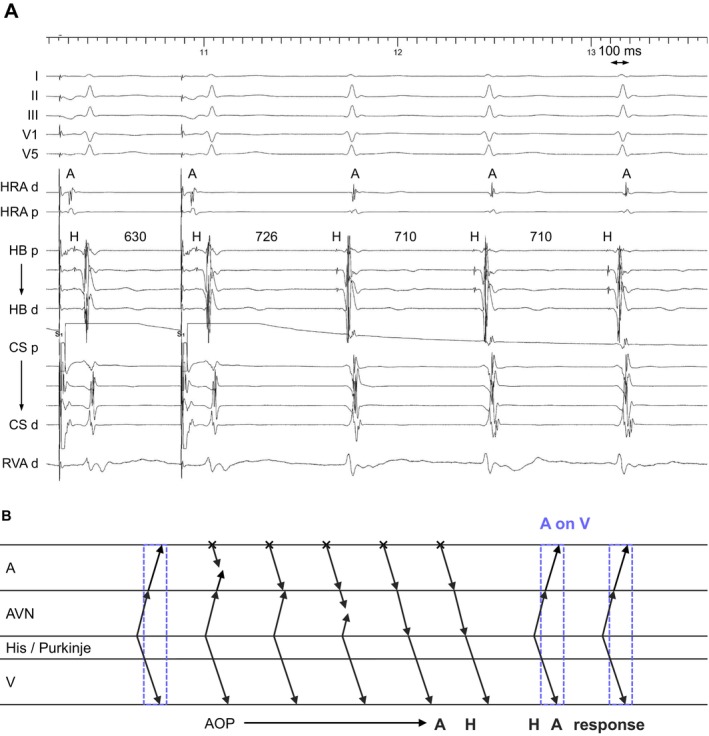
(A) Intracardiac electrograms and (B) schematic laddergram of atrial overdrive pacing (AOP) during junctional tachycardia (JT) illustrating the A on V mechanism and an A‐H‐H‐A response. This laddergram is not intended to exactly match the intracardiac electrograms shown in (A). Abbreviations are as in Figure 1.

**FIGURE 3:**
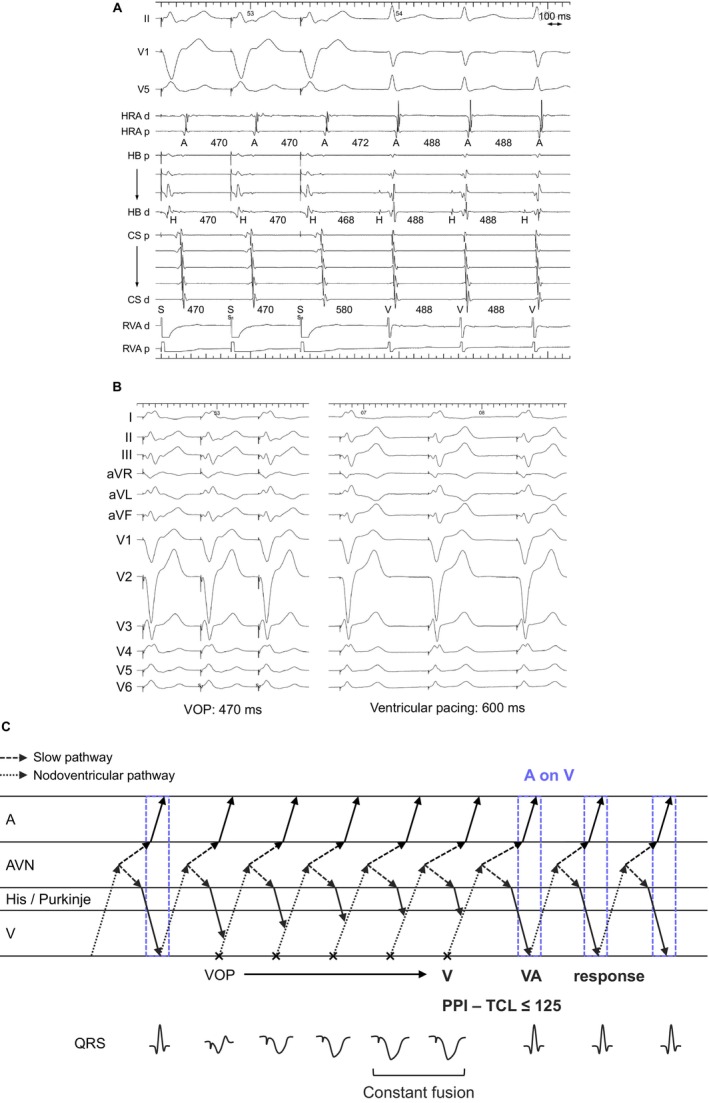
(A) Intracardiac electrograms during ventricular overdrive pacing (VOP) in orthodromic reciprocating tachycardia via a nodoventricular pathway. (B) Twelve‐lead ECGs during VOP and during ventricular pacing in sinus rhythm. Constant QRS fusion is observed. (C) Schematic laddergram of VOP illustrating the A‐on‐V mechanism with a V‐V‐A response. This laddergram is not intended to exactly match the intracardiac electrograms shown in (A). Abbreviations are as in Figure 1.

**FIGURE 4:**
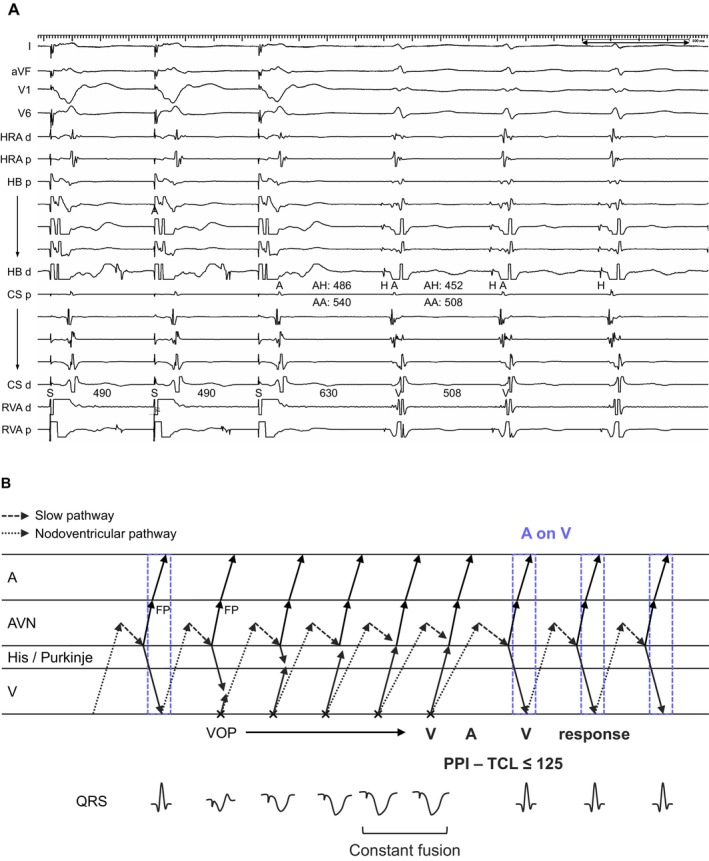
(A) Intracardiac electrograms during ventricular overdrive pacing (VOP) in orthodromic reciprocating tachycardia via a nodoventricular pathway. The uncorrected and corrected post‐pacing interval minus tachycardia cycle length are 122 and 88 ms, respectively. (B) Schematic laddergram of VOP illustrating the A‐on‐V mechanism with a V‐A‐V response. This laddergram is not intended to exactly match the intracardiac electrograms shown in (A). Abbreviations are as in Figure 1.

**FIGURE 5:**
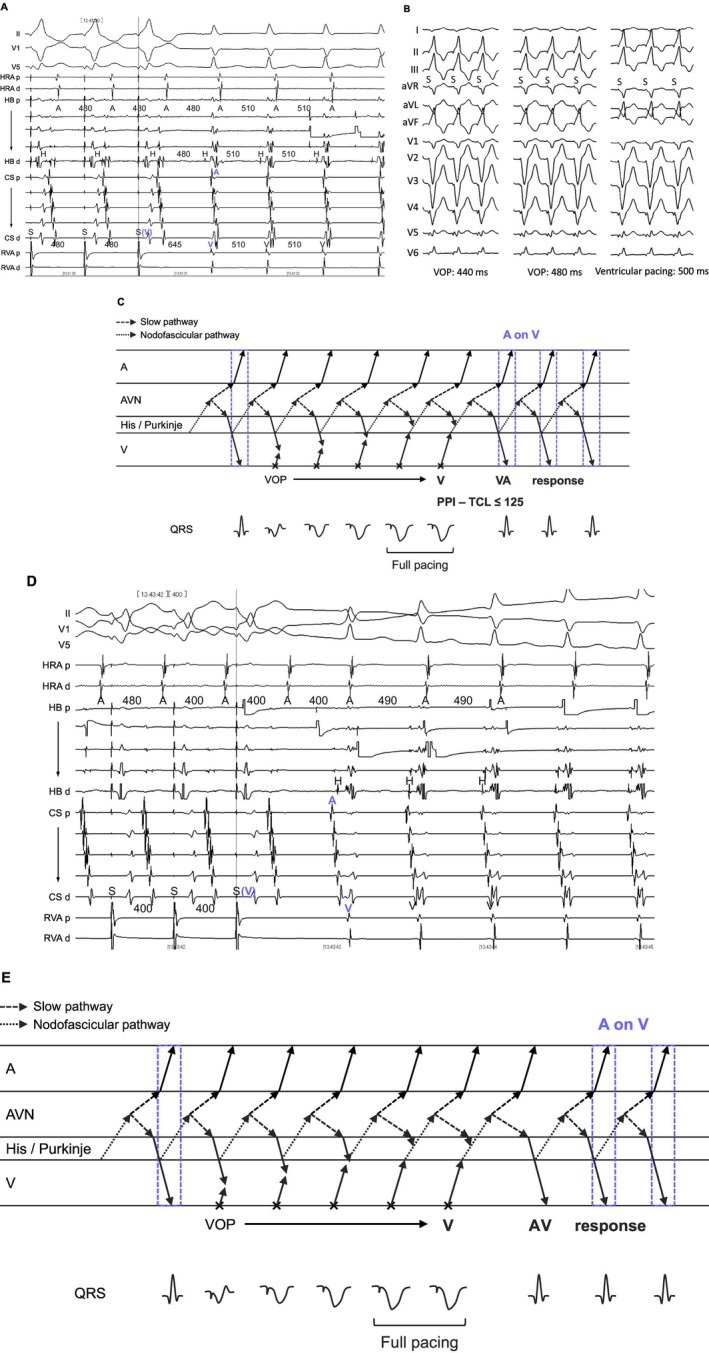
(A) Intracardiac electrograms during ventricular overdrive pacing (VOP) at a pacing cycle length (PCL) of 480 ms in orthodromic reciprocating tachycardia via a nodofascicular pathway. (B) Twelve‐lead ECGs during VOP at PCLs of 480 and 440 ms, and during ventricular pacing in sinus rhythm, demonstrating the absence of QRS fusion. (C) Schematic laddergram of VOP at a PCL of 480 ms illustrating the A‐on‐V mechanism with a V‐V‐A response. (D) Intracardiac electrograms during VOP at a PCL of 400 ms. (E) Schematic laddergram of VOP at a PCL of 400 ms illustrating the V‐A‐V response. The laddergrams in (C) and (E) are not intended to exactly match the intracardiac electrograms shown in (A) and (D), respectively. Abbreviations are as in Figure 1. PPI = post‐pacing interval, TCL = tachycardia cycle length.

**FIGURE 6:**
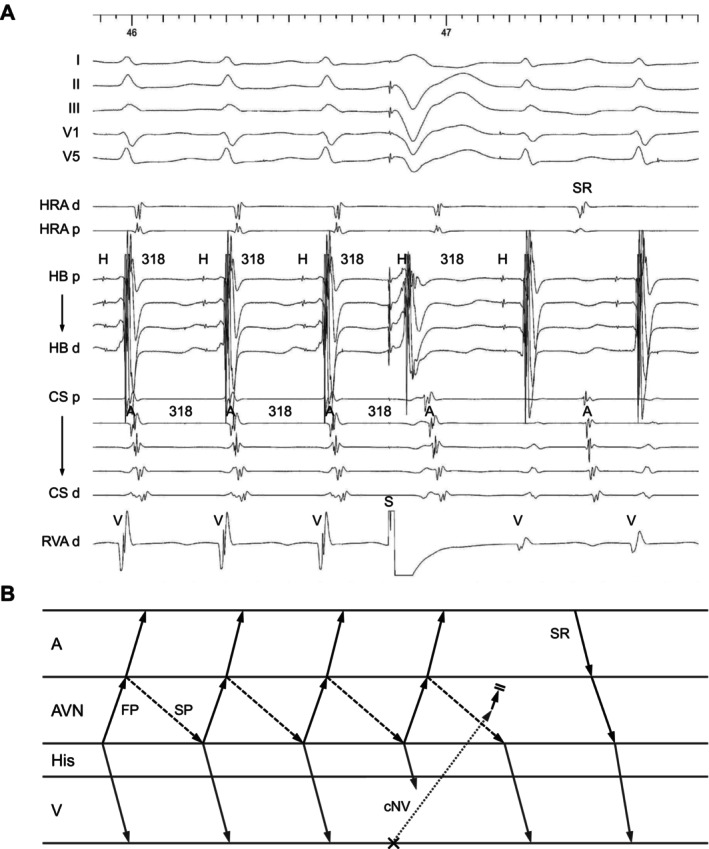
(A) Intracardiac electrograms and (B) schematic laddergram of slow‐fast AVNRT with a bystander concealed nodoventricular (cNV) pathway inserting into the retrograde limb (fast pathway). A His‐refractory PVC terminates the tachycardia one cycle later without atrial capture. Abbreviations are as in Figure 1. SR = sinus rhythm.

Among a total of 1484 patients with A on V tachycardia, 24 cases (1.6%) had diagnoses other than conventional AVNRT: AT (*n* = 7, Figure 1), JT (*n* = 6, Figure 2), ORT via NV (*n* = 6, Figures 3 and 4), or NF (*n* = 1, Figure 5) pathway, and AVNRT with a bystander NV pathway (*n* = 4, Figure 6) (Table 1). The mean AH and HV intervals during sinus rhythm were 93 ± 18 and 42 ± 9 ms, respectively. During tachycardia, the TCL was 423 ± 116 ms, with HA and AH intervals of 48 ± 16 and 378 ± 118 ms, respectively. The H‐A‐V sequence was observed in 12 cases (50%) and the H‐V‐A sequence in 12 cases (50%).

**TABLE 1:** Baseline and electrophysiologic characteristics of the patients.

	*n* = 24
Age (years)	52 ± 18
Man	9 (33)
Baseline measurements	
AH interval (ms)	93 ± 18
HV interval (ms)	42 ± 9
Tachycardia features	
TCL (ms)	423 ± 116
HA interval (ms)	48 ± 16
AH interval (ms)	378 ± 118
H‐A‐V sequence	12 (50)
H‐V‐A sequence	12 (50)
Diagnosis	
Atrial tachycardia	7 (29)
Junctional tachycardia	6 (25)
Orthodromic reciprocating tachycardia via a nodoventricular pathway	6 (25)
Orthodromic reciprocating tachycardia via a nodofascicular pathway	1 (4)
Atrioventricular nodal reentrant tachycardia with a bystander nodoventricular pathway	4 (17)

*Note:* Values are shown as the mean ± SD or *n* (%).

Abbreviations: AH, atrio‐his; HA, his‐atrial; HV, his‐ventricular; TCL, tachycardia cycle length.

### 3.2 AT

Of the 7 AT cases, AT originated from the crista terminalis (*n* = 4) or para‐Hisian area (*n* = 3), diagnosed by V‐A‐A‐V response after VOP (*n* = 3) or absence of VA linking after differential AOP (*n* = 6, Figure 1) (Table 2). TCL and HA intervals were 354 ± 68 and 46 ± 19 ms, respectively. The H‐A‐V sequence was observed in 3 cases (43%) and the H‐V‐A sequence in 4 cases (57%).

Dual AV nodal physiology, characterized by a jump in the AH interval, was noted in 5 cases (71%), AH interval during AT was 304 ± 65 ms. These findings suggest that most ATs were conducted to the ventricle over the slow pathway (SP). In the remaining two patients without an AH jump, the AH interval was 90 and 75 ms during sinus rhythm, but prolonged to 246 and 216 ms, respectively, during AT.

**TABLE 2:** Electrophysiologic characteristics and diagnostic pacing responses in non‐AVNRT A on V tachycardias.

Diagnosis	AT (*n* = 7)	JT (*n* = 6)	NV‐ORT (*n* = 6)	NF‐ORT (*n* = 1)	AVNRT + NV (*n* = 4)
Antegrade dual AV nodal physiology	5 (71)	6 (100)	5 (83)	0 (0)	4 (100)
Tachycardia features					
TCL (ms)	354 ± 68	523 ± 130	412 ± 115	505	388 ± 89
HA interval (ms)	46 ± 19	41 ± 13	47 ± 11	70	63 ± 6
AH interval (ms)	304 ± 65	482 ± 125	365 ± 123	435	325 ± 90
H‐A‐V sequence	3 (43)	5 (83)	3 (50)	1 (100)	0 (0)
H‐V‐A sequence	4 (57)	1 (17)	3 (50)	0 (0)	4 (100)
Diagnostic pacing maneuvers					
Tachycardia reset after His‐refractory PVC	0/3 (0)	0/3 (0)	5 (83)	1 (100)	3 (75)
Ventricular overdrive pacing					
VA dissociation	4 (57)	0/3 (0)	1 (17)	0 (0)	0 (0)
V‐A‐A‐V response	3/3 (100)	0/3 (0)	0 (0)	0 (0)	0 (0)
V‐A‐V response	0/3 (0)	3/3 (100)	1 (17)	0 (0)	0 (0)
V‐V‐A response	0/3 (0)	0/3 (0)	3 (50)	1 (100)	4 (100)
PPI—TCL (ms)	309 ± 108	154 ± 5	94 ± 29	137	159 ± 17
Corrected PPI—TCL (ms)	247 ± 118	106 ± 8	83 ± 23	127	146 ± 24
Orthodromic his capture	0/5 (0)	0/3 (0)	4 (67)	1 (100)	0 (0)
Presence of QRS fusion	0 (0)	0 (0)	6 (100)	0 (0)	0 (0)
Diagnosis of JT with atrial extrastimulus	—	4/4 (100)	—		—
Atrial overdrive pacing					
A‐H‐A response	6/6 (100)	0/5 (0)	2/2 (100)	—	—
A‐H‐H‐A response	0/6 (0)	5/5 (100)	0/2 (0)	—	—
Absence of VA linking	6/6 (100)	—	0/2 (0)	—	—
Origin of AT					
crista terminalis	4 (57)	—	—	—	—
para‐hisian region	3 (43)	—	—	—	—

*Note:* Values are shown as the mean ± SD or *n* (%).

Abbreviations: AH, atrio‐His; AT, atrial tachycardia; AV, atrioventricular; AVNRT + NV, atrioventricular nodal reentrant tachycardia with a bystander nodoventricular pathway; HA, His‐atrial; JT, junctional tachycardia; NF, nodofacicular; NV, nodoventricular; ORT, orthodromic reciprocating tachycardia; PPI, post‐pacing interval; PVC, premature ventricular contraction, TCL, tachycardia cycle length; VA, ventriculoatrial.

### 3.3 JT

Of the 6 JT cases, JT was diagnosed on the basis of findings during atrial extrastimulus (*n* = 4) and an A‐H‐H‐A response following AOP (*n* = 5, Figure 2) (Table 2). TCL and HA intervals were 523 ± 130 and 41 ± 13 ms, respectively. The H‐A‐V sequence was observed in 5 cases (83%) and the H‐V‐A sequence in 1 case (17%).

### 3.4 NV/NF‐ORTs

Of the 6 NV‐ORTs (Figures 3 and 4) and 1 NF‐ORT (Figure 5), ORT was diagnosed on the basis of the combination of the following findings: tachycardia resetting following His‐refractory PVC (*n* = 6), orthodromic His capture (*n* = 5), a V‐V‐A response (*n* = 4, Figures 3A,C, 5A,C), PPI − TCL ≤ 125 ms (*n* = 4), and corrected PPI − TCL ≤ 110 ms upon cessation of VOP (*n* = 4), and paradoxical AH response during AOP (*n* = 1) (Table 2). All NV‐ORTs and 1 NF‐ORT met more than one (3 ± 1) of the observations above. The PPI − TCL and corrected PPI − TCL intervals were 94 ± 29 and 83 ± 23 ms in NV‐ORT, and 137 and 127 ms in NF‐ORT, respectively. All NV/NF pathways were presumed to connect to the SP.

NV‐ORT and NF‐ORT were differentiated on the basis of the presence of QRS fusion during VOP. In NV‐ORT, the TCL and HA intervals were 412 ± 115 and 47 ± 11 ms, respectively. The H‐A‐V sequence was observed in 3 cases (50%) and the H‐V‐A sequence in 3 cases (50%). In NF‐ORT, the TCL and HA intervals were 505 and 70 ms, respectively. The H‐A‐V sequence was observed.

Given the V‐V‐A response observed in 3 NV‐ORT and 1 NF‐ORT cases, the accurate HA interval during ORT was 503 ± 119 ms, which exceeded the TCL in these cases. This response suggests that the A on V pattern in these cases arises from atrial activation coinciding with the ventricular activation of the following beat (Figures 3C and 5C). Because NV/NF pathways often connect to the SP, the long HA interval may reflect the sum of retrograde conduction over the NV/NF pathway and through the SP. During ORT, conduction over the NV/NF pathway enters the SP at its insertion site, conducting retrogradely through the proximal SP to the atrium while simultaneously propagating anterogradely through the distal SP toward the His bundle as the next beat.

However, a V‐A‐V response was observed in 1 ORT (Figure 4), whereas in the remaining cases, the response was indeterminate because of VA dissociation or tachycardia termination during VOP. Furthermore, in two NV/NF‐ORT cases that initially exhibited a V‐V‐A response, VOP at a shorter PCL revealed a V‐A‐V response (Figure 5D,E). This V‐A‐V response was classified with 2 types: short SA interval (Figure 4) and extremely long SA interval (Figure 5D,E). The first could be explained by the presence of a retrograde fast pathway. During VOP and even during tachycardia, the atrium may be activated via retrograde conduction over the His–Purkinje system and the fast pathway, which results in a very short SA interval and a V‐A‐V response (Figure 4). Even in the absence of a retrograde fast pathway, differences in the decremental properties of the SP proximal and distal to the NV/NF attachment may account for a V‐A‐V response. Specifically, if decremental conduction is less pronounced in the proximal SP (retrograde) than in the distal SP (anterograde), conduction over the NV/NF pathway during VOP may reach the ventricle through the distal SP and His–Purkinje system before it reaches the atrium via the proximal SP, thereby generating a V‐A‐V response (Figure 5D,E).

### 3.5 AVNRT With a Bystander NV Pathway

Of the 4 AVNRT cases with a bystander NV pathway, tachycardia resetting was observed following a His‐refractory PVC in all cases (Table 2). In 2 cases, the tachycardia was delayed immediately after the His‐refractory PVC; however, this resetting phenomenon disappeared after delivery of an early PVC. On the basis of the reset sequence, these NV pathways were considered to insert into the anterograde limb of the AVNRT circuit. In the remaining 2 cases, tachycardia was delayed (*n* = 1) or terminated without atrial capture (*n* = 1) one cycle after delivery of a His‐refractory PVC (Figure 6). According to the reset sequence, these NV pathways were presumed to insert into the retrograde limb of the AVNRT circuit. The TCL and HA intervals were 523 ± 130 and 41 ± 13 ms, respectively. All cases demonstrated an H‐V‐A sequence. PPI − TCL and corrected PPI − TCL upon cessation of VOP were 159 ± 17 and 146 ± 24 ms, respectively. Orthodromic His capture was not observed in any case. Tachycardia was rendered non‐inducible by conventional slow pathway ablation in all cases.

## 4 Discussion

### 4.1 Main Findings

On the basis of these results, although conventional AVNRT accounted for the vast majority of A on V tachycardia, 24 of 1484 cases (1.6%) were attributable to alternative diagnoses: AT (*n* = 7) with predominant anterograde conduction over the SP, JT (*n* = 6), ORT via an NV (*n* = 6) or NF (*n* = 1) pathway, and AVNRT with a bystander NV pathway (*n* = 4) (Table 2). These findings underscore that, although an A on V tachycardia is strongly suggestive of AVNRT, it is not exclusive to this mechanism. Therefore, careful diagnostic pacing maneuvers are required for an accurate diagnosis. These arrhythmias can be categorized into two types of A on V tachycardia. The first is a true A on V tachycardia, in which the atrium and ventricle are activated simultaneously within the same beat. The second is a pseudo‐A on V tachycardia, where the atrial and ventricular activations appear to be in the same beat, but in fact represent activations from different beats that happen to overlap in timing.

### 4.2 True A on V Tachycardia

The true A on V tachycardia includes AVNRT (slow‐fast type or slow‐slow type with a lower common pathway) with/without a bystander NV pathway and JT. The diagnostic maneuvers have been well developed. The true A on V tachycardia can occur when the origin or reentrant circuit is confined to the atrioventricular node. The laddergrams of these tachycardias are shown in Figure 2B and Central illustration. Although JT is rare in the adult population undergoing SVT ablation, its presence within the A on V tachycardia cohort illustrates the importance of maintaining diagnostic vigilance, particularly when electrogram findings are ambiguous.

### 4.3 Pseudo‐A on V Tachycardia

The pseudo‐A on V tachycardia includes ATs and ORTs via a NV/NF pathway. In these tachycardias, atrial and ventricular activations may appear to occur within the same cardiac cycle; however, they actually represent activations from different beats that overlap in timing.

Among ATs presenting with an A on V sequence, a substantial proportion demonstrated the presence of an anterograde SP, with a markedly prolonged AH interval during tachycardia (304 ± 65 ms). In the seven cases analyzed, the tachycardia origin was either the crista terminalis (remote from the AV node) or the para‐Hisian region (adjacent to the AV node). These findings suggest that the A on V pattern in AT is attributable to anterograde conduction over the SP, rather than solely the anatomical distance between the AT focus and the AV node. The prolonged AH interval results in atrial activation that temporally overlaps with the ventricular activation of the preceding beat (Figure 1C and Central illustration). Correct diagnosis was confirmed through hallmark pacing responses, such as a V‐A‐A‐V sequence following VOP and the absence of VA linking during AOP. These findings emphasize the diagnostic utility of pacing maneuvers in distinguishing AT from AVNRT.

In contrast, 70% of ORTs via an NV/NF pathway exhibited another type of pseudo‐A on V tachycardia, characterized by a V‐V‐A response after VOP. In this setting, atrial activation overlapped with the ventricular activation of the subsequent beat, producing the appearance of an A on V sequence (Figures 3C and 5C, and Central illustration). This pattern is likely related to the anatomical connection of NV/NF pathways to the SP, whereby the atrium is activated retrogradely through the proximal SP, whereas the same impulse propagates anterogradely through the distal SP toward the His bundle as the next beat. Interestingly, some NV/NF‐ORT cases demonstrated a conventional V‐A‐V response (Figures 4 and 5D,E). These findings indicate that NV/NF‐ORT can manifest as true A‐on‐V tachycardia only when retrograde conduction over a fast pathway shortens the SA interval, possibly because a change in autonomic tone caused a spontaneous shift to fast pathway conduction. In the absence of a retrograde fast pathway, both V‐V‐A and V‐A‐V responses should be regarded as pseudo‐A on V tachycardia, reflecting the markedly prolonged conduction time over the retrograde SP.

To our knowledge, no previous reports have described AVNRT cases exhibiting a V‐V‐A response. A V‐V‐A response strongly indicates that the atrium is not part of the tachycardia circuit. Although whether the atrium is incorporated into the AVNRT circuit remains controversial, a V‐V‐A response is theoretically incompatible with AVNRT if the atrium is required for the circuit. Even under the alternative hypothesis in which the atrium is not part of the AVNRT circuit but is connected via an upper common pathway, the occurrence of a V‐V‐A response would be highly unlikely, because in the setting of slow‐fast AVNRT with an upper common pathway, the SA interval through the retrograde fast pathway and the upper common pathway would theoretically not be able to exceed the TCL.

### 4.4 Differentiation of NV‐ORT and NF‐ORT

Differentiation between NV‐ORT and NF‐ORT remains an important but challenging issue in clinical practice. Traditionally, the distinction relies on the conventional observation of QRS fusion during VOP, with the presence of constant or progressive fusion favoring NV‐ORT and its absence suggesting NF‐ORT. In our cohort, both the uncorrected and corrected PPI–TCL appeared to be longer in NF‐ORTs compared with NV‐ORTs. This observation is consistent with the expected circuit anatomy: in NF‐ORT, the reentrant circuit is confined to the AV node, His–Purkinje system, and the NF pathway, with the ventricle lying outside the circuit. Consequently, pacing from the ventricle introduces additional conduction time before reentry into the circuit, leading to a longer PPI after VOP. In contrast, in NV‐ORT, the ventricle forms part of the reentrant pathway, allowing earlier engagement of the circuit and resulting in a shorter PPI.

### 4.5 The Clinical Implications

The clinical implications of these findings are threefold. First, although AVNRT remains the predominant mechanism underlying A‐on‐V tachycardia, electrophysiologists should recognize that approximately 1.6% of cases may involve alternative mechanisms, which require tailored diagnostic and therapeutic strategies. Second, exclusive reliance on electrogram characteristics—particularly the A on V pattern or HA interval—may lead to misdiagnosis, particularly in the presence of dual AV nodal physiology or atypical conduction pathways. A systematic approach incorporating diagnostic pacing maneuvers is therefore essential for accurate diagnosis and effective ablation. Third, since A‐on‐V tachycardia proves to be AVNRT in 98.4% of cases, and given that even in NV/NF‐ORT, the slow pathway remains the appropriate ablation target, when encountering a patient with clinical A‐on‐V tachycardia in whom tachycardia cannot be induced in the EP lab, empiric slow pathway ablation may be considered.

### 4.6 Study Limitations

Our results must be interpreted in light of our study limitations. The study was conducted as a small, retrospective investigation. Nonetheless, we believe that our findings are clinically meaningful because they are based on meticulous analysis of the electrophysiologic characteristics of the arrhythmias and of the electrocardiographic measurements. In addition, the choice of diagnostic pacing maneuvers depended on the institution's or physician's preference, although VOP was performed in all cases. The lack of standardization in these diagnostic strategies may have introduced variability, and future standardization across centers could help facilitate interstudy comparisons and improve the accuracy and reproducibility of electrophysiological diagnoses. Our electrophysiologic findings were not supported by the histological evidence. Further histological study would be warranted.

## 5 Conclusions

Although conventional AVNRT accounts for the vast majority of A on V tachycardia, a small subset is attributable to alternative mechanisms, including AT, JT, and ORT, via an NV/NF pathway, and AVNRT with a bystander NV pathway. These findings demonstrate that an A on V pattern is not specific to conventional AVNRT and support a systematic diagnostic approach incorporating pacing maneuvers to accurately define the underlying mechanism (Table 2).

## Ethics Statement

The review board of Nihon University Itabashi Hospital and the review board of each participating center approved the data collection and analysis.

## Consent

Patients had consented to the use of their data for research purposes through an opt‐out method.

## Conflicts of Interest

The authors declare no conflicts of interest.

## Data Availability

Research data are not shared.
